# SARS-CoV-2 Virus−Host Interaction: Currently Available Structures and Implications of Variant Emergence on Infectivity and Immune Response

**DOI:** 10.3390/ijms221910836

**Published:** 2021-10-07

**Authors:** Luís Queirós-Reis, Priscilla Gomes da Silva, José Gonçalves, Andrea Brancale, Marcella Bassetto, João R. Mesquita

**Affiliations:** 1 Abel Salazar Institute of Biomedical Sciences (ICBAS), University of Porto, 4050-313 Porto, Portugal; up201205115@up.pt (L.Q.-R.); priscilla@ua.pt (P.G.d.S.); 2 Epidemiology Research Unit (EPIunit), Institute of Public Health, University of Porto, 4050-091 Porto, Portugal; 3 LEPABE—Laboratory for Process Engineering, Environment, Biotechnology and Energy, Faculty of Engineering, University of Porto, 4200-465 Porto, Portugal; 4 Institute of Sustainable Processes, University of Valladolid, 47011 Valladolid, Spain; zemcg5@gmail.com; 5 Cardiff School of Pharmacy and Pharmaceutical Sciences, Cardiff University, Cardiff CF10 3NB, UK; brancalea@cardiff.ac.uk; 6 Department of Chemistry, Faculty of Science and Engineering, Swansea University, Swansea SA2 8PP, UK; marcella.bassetto@swansea.ac.uk

**Keywords:** SARS-CoV-2, Spike protein, hACE2, protein structures

## Abstract

Coronavirus disease 19, or COVID-19, is an infection associated with an unprecedented worldwide pandemic caused by the Severe Acute Respiratory Syndrome Coronavirus 2 (SARS-CoV-2), which has led to more than 215 million infected people and more than 4.5 million deaths worldwide. SARS-CoV-2 cell infection is initiated by a densely glycosylated spike (S) protein, a fusion protein, binding human angiotensin converting enzyme 2 (hACE2), that acts as the functional receptor through the receptor binding domain (RBD). In this article, the interaction of hACE2 with the RBD and how fusion is initiated after recognition are explored, as well as how mutations influence infectivity and immune response. Thus, we focused on all structures available in the Protein Data Bank for the interaction between SARS-CoV-2 S protein and hACE2. Specifically, the Delta variant carries particular mutations associated with increased viral fitness through decreased antibody binding, increased RBD affinity and altered protein dynamics. Combining both existing mutations and mutagenesis studies, new potential SARS-CoV-2 variants, harboring advantageous S protein mutations, may be predicted. These include mutations S13I and W152C, decreasing antibody binding, N460K, increasing RDB affinity, or Q498R, positively affecting both properties.

## 1. Introduction

Coronavirus disease 19, or COVID-19, is an infection associated with an unprecedented global pandemic caused by the severe acute respiratory syndrome coronavirus 2 (SARS-CoV-2), which resulted in more than 215 million infected people and more than 4.5 million deaths [1,2,3]. SARS-CoV-2 was first identified in December 2019 in Wuhan, Hubei province of China, in individuals exposed at a seafood/wet market and was successfully isolated and sequenced in January 2020 [1]. Genetic evidence suggests that the virus originates from animals, particularly bats, as SARS-CoV-2 has 96% sequence identity to a strain found in bats (BatCov RaTG13), possibly having pangolins, minks, turtles, or snakes as the intermediate host [1,4]. 

**Figure 1:**
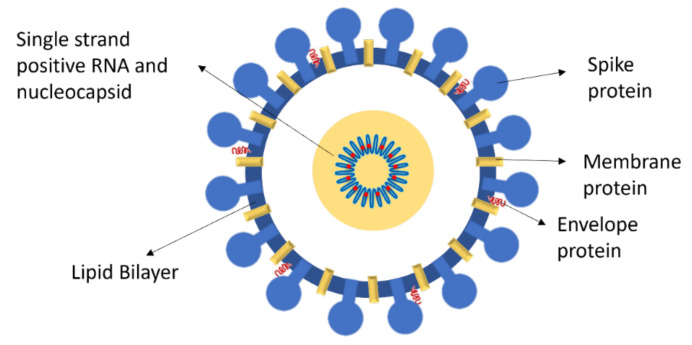
Representation of SARS-CoV-2 main structural proteins.

Coronaviruses (CoVs) are a group of RNA viruses belonging to the subfamily *Orthocoronaviridae* that cause disease in a variety of domestic and wild animals (e.g. porcine, bovine, feline and avian, and bat and whale strains) [5]. A defining characteristic in all CoVs is the crown-like viral particle, from which their name is derived [6,7]. It consists of three major structural proteins (spike, membrane, and envelope), that protrude from the viral envelope and have a critical function in cell recognition and fusion [6]. Additionally, the nucleocapsid (protein N) binds and stabilizes the viral genome (Figure 1) [8].

Furthermore, they have the second largest RNA viral genome, a positive sense genome of single-stranded RNA (+ssRNA), with an average size of 30 kb [9]. Importantly, CoVs have a large genome with a high mutation rate (one nucleotide per 1000 to 10,000 nucleotides replicated), associated with the RNA dependent RNA polymerase, and a random template switching during RNA duplications, that causes a high frequency of homologous RNA recombination [5]. These characteristics are responsible for the special plasticity of CoVs when it comes to accommodating and modifying genes, explaining the broad range of hosts infected by them [5].

Taken together, these factors have led to a diversity of strains and genotypes highly adaptable to new hosts and ecological niches, causing major zoonotic outbreaks [5]. 

SARS-CoV-2 is transmitted via aerosols or droplets, typically within one metre or in poorly ventilated and/or crowded indoor settings, and possibly by touching contaminated and eyes, nose, or mouth, with both symptomatic and asymptomatic patients being the main source of infection [1,10]. The immune response appears to be characteristic of each individual, with a unique collection of antibodies (Abs) and varying efficacy of viral neutralization potency observed [11].

Over time, numerous SARS-CoV-2 variants have been reported, differing by one or more mutations from the first virus sequenced, the so-called original virus variant, which corresponds to the Wuhan strain [12]. Some of these have been classified as variants of concern (VOC), defined by their features, such as increased transmissibility, virulence and disease severity, decreased Ab neutralization or vaccine efficiency and/or errors in current detection protocols [13,14,15]. The list of variants of concern is frequently updated according to new information, by the Centers for Disease Control and Prevention (CDC), World Health Organization or the European Centre for Disease Prevention and Control (ECDC) [14,15,16,17,18]. Additionally, some variants are classified as variants of interest (VOI), characterized by mutations with predicted negative features, although with insufficient evidence, by limited prevalence or epidemiological data suggesting an emerging risk to public health [14,15,16]. Variants can also be de-escalated, particularly if they cease to circulate, if, despite circulating, they have a negligible effect on the epidemiological situation or if scientific evidence shows no concerning features (Table 1) [14].

### Glycoprotein: Cell Recognition and Infection

As with all CoVs, SARS-CoV-2 cell infection is initiated by a densely glycosylated spike (S) protein, a trimeric class I fusion protein that binds to human angiotensin converting enzyme 2 (hACE2), acting as the functional receptor [1]. hACE2 expression is ubiquitous in the human body and although the degree of expression varies between organs, it is at its highest in type II alveolar epithelial cells in the lungs, which indicates the primary target for the virus [27]. In addition, hACE2 expression is also high in other tissues, such as myocardial cells, kidney and enterocytes, which explains less common symptoms as well as the multiorgan injuries observed in severe cases [27].

**Figure 2:**
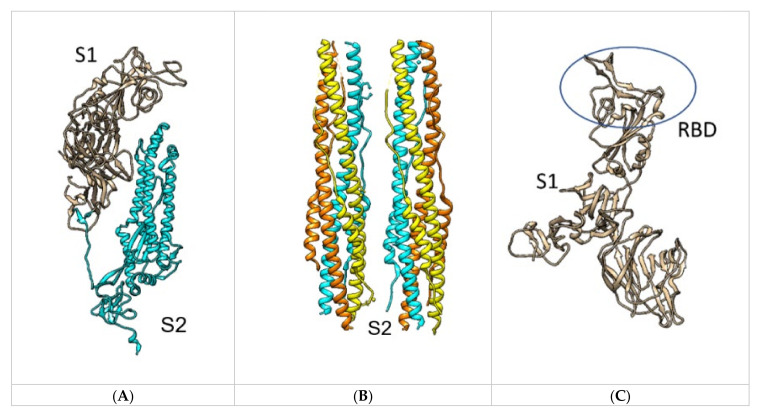
(**A**) Single chain in trimeric spike protein with subunit S1 (Tan) and S2 (Cyan) in 7A94. (**B**) Post-fusion conformation of all S2 units in the trimeric spike in 6LXT. (**C**) S1 subunit, with the RBD highlighted in 7A94 [29].

In general, the S protein exists in two main conformations, prefusion and post-fusion, requiring major structural rearrangements after recognition to promote cell fusion [28]. The S protein consists of two subunits (S1 and S2) responsible for cell recognition and membrane fusion, respectively [1]. Although S1 and S2 are originally covalently bound, there is a cleavage site between the subunits, where host serine proteases, such as furin, transmembrane protease serine protease 2 (TMPRSS2) or cathepsin L, cleave the protein [1,11,28]. Notably, TMPRSS2 is co-expressed with hACE2 in nasal epithelial cells, lungs and bronchial branches [11]. When the S1 and S2 subunits are cleaved, the protein is activated and can undergo the necessary conformational changes to achieve the post-fusion conformation and consequently enable cell infection (Figure 2) [1,28].

The S1 subunit formed of the N-terminal domain (NTD) and a receptor binding domain (RBD) that directly interacts with hACE2. Therefore, it is considered a key target for antivirals and neutralizing Abs, accounting for 90% of the neutralizing activity in convalescent sera [1,30,31,32]. Despite the low percentage of NTD-directed Abs, some are still potent inhibitors [32]. The NTD and two neighbouring regions, namely a β-hairpin (residues 140-158) and a loop (residues 245-264), are collectively termed an antigenic supersite, where NTD directed Abs bind [32]. The NTD-directed Abs neutralizing activity is due to both viral entry blocking and also limiting cell-to-cell spread of the virus [32].

Regarding the RBD, it can be further divided in two structural domains, a highly conserved core, and a variable external subdomain that binds hACE2 [1]. 

Subunit S2 contains two heptad repeats, a transmembrane domain, a cytoplasmic tail, a central helix, and the fusion peptide that is critical for the main function of S2 of merging the host and viral cell membranes (Figure 2) [31]. Additionally, N-linked glycans are extensively present along the molecule and are essential in proper folding [1,31].

**Figure 3:**
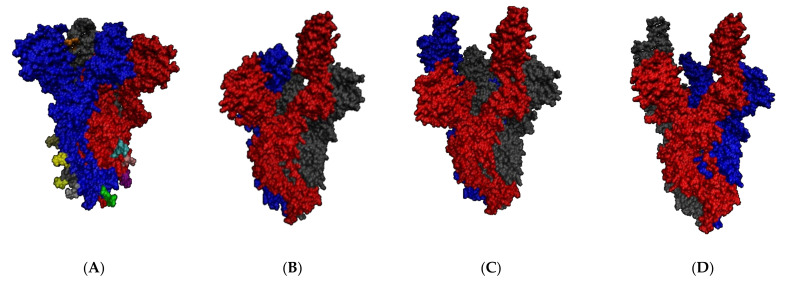
Spike protein conformations, with each spike monomer is represented by a colour (blue, red, gray). (**A**) All RBD down (PBD 6VVX) (**B**) 1 RBD up (PDB 7A94) (**C**) 2 RBD up (PDB 7A97) (**D**) All RBD up (PDB 7A98) [33].

Even if the S protein is activated through the cleavage process, the RBDs can still be inaccessible for interaction [28]. Each RBD can be either down (closed conformation), inaccessible for interaction, or up (open conformation). The open conformation is the necessary state for recognition, with the recognition motifs protruding from the S protein (Figure 3) [1,28].

To engage the host cell receptor, the RBD undergoes hinge-like conformational changes, transiently switching from a down conformation, to a less stable up conformation. Each RBD is capable of individually changing its conformation (Figure 3) [28]. When ACE2 interacts with the RBD, it binds tightly and stabilizes the up conformation [1,34].

In this review, the interaction of hACE2 with the RBD is explored, namely the important contacts between proteins and how this interaction can be modulated to increase affinity or reduce Ab binding. Additionally, we will also try to elucidate how the binding of hACE2 affects S1/S2 interaction, the importance of this interaction in cell fusion and how mutations can influence infectivity and immune response. This information may be important to predict or explain the changes in infectivity or immune response for new variants, which has been studied for the SARS-CoV-2 Delta variant, currently becoming the most prevalent variant worldwide [35].

**Figure 4:**
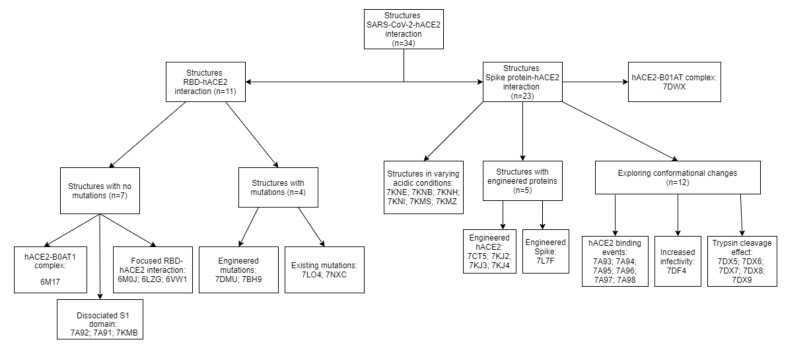
Organizational scheme of all structures found in this review. Molecular graphics analyses were performed with UCSF Chimera and Visual molecular dynamics [29,33]. A homology model for the S protein of SARS-CoV-2 Delta variant was created using SWISS-MODEL [37].

A total of 37 structures were selected, three of which are complexes that, despite involving the SARS-CoV-2 S protein, also have an Ab or non-human ACE2, and therefore would not reliably represent the critical interactions between the virus and the human protein. The remaining structures can be broadly divided into two categories: studying the RBD-hACE2 interaction or full spike protein behaviour, focusing on the conformation changes from recognition to fusion (Figure 4) [21]. Furthermore, some structures also have hACE2 in a complex with broad neutral amino acid transporter 1 (B0AT1), which stabilizes hACE2 homodimers [36].

## 2. Discussion

### 2.1. SARS-CoV-2 Spike RBD – hACE2 Interaction

#### 2.1.1. Structures with No Mutation

The RBD/hACE2 interaction structures represent the domains of both proteins that contribute to this interaction, providing the basis for computational protein-protein interaction studies, mutation analysis, drug design and computational models of the full spike protein, as it is harder to maintain a similar resolution for significantly larger structures [38,39,40,41].

**Figure 5:**
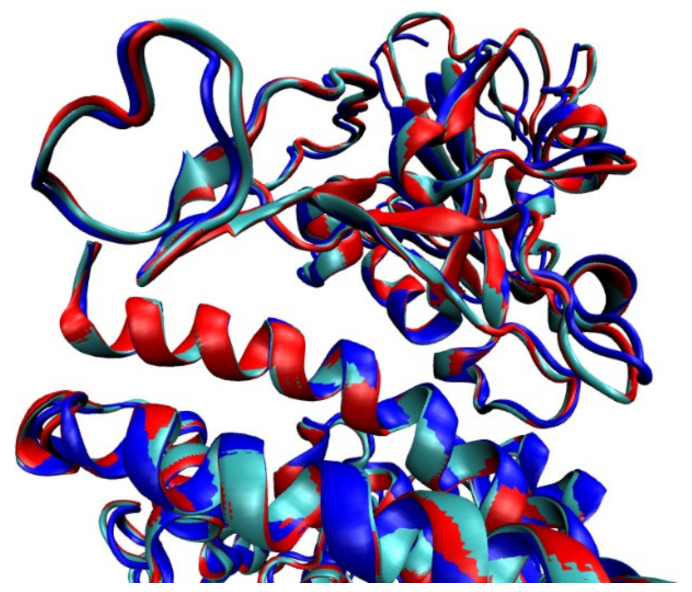
Alignment and superposition of the models representing the interaction with no mutations (PDB 6VW1/6M0J/6LZG) [33].

The structures used for these analyses represent the wild-type SARS-CoV-2 S protein/ACE2 interaction without mutations (PDB 6VW1/6M0J/6LZG), with an Root Mean Square Distance (RMSD) of 0.32 Å between them (Figure 5) [39,42]. 

Consistently, in all structures focusing on this interaction in wild-type SARS-CoV-2 S protein, the contact interface is divided into two clusters at both ends of the α1 helix in hACE2, leaving a central cavity filled with water [40,43]. In these sections, there are over 10 H-bonds, multiple salt bridges, π-stacking and non-polar interactions [36,40,43].

**Figure 6:**
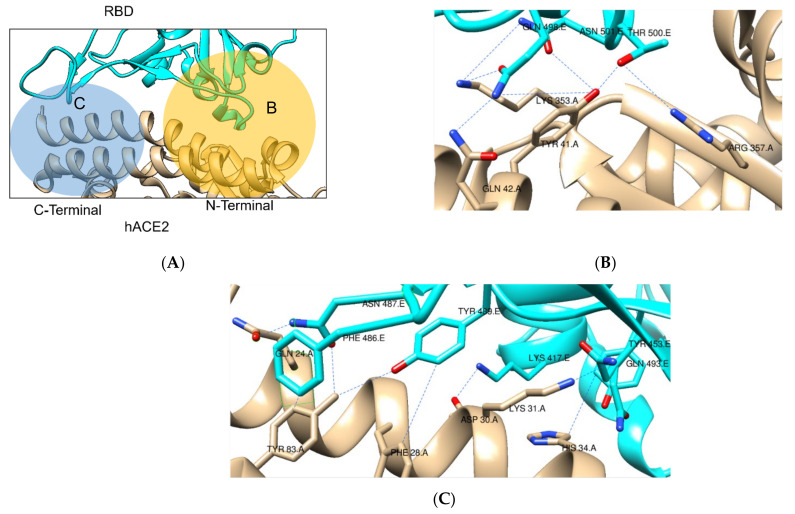
Interface between SARS-CoV-2 S protein RBD (cyan) and hACE2 (tan), in the S protein/ACE2 interaction without mutations structure (PDB 6M0J). (**A**) Full view of RBD—hACE2 interface (**B**) N-terminal portion of the α1 helix with a network of H-bonds (**C**) C-terminal interactions, with π-stacking interactions, h-bonds and Van der Waals forces [29].

In the N-terminal portion of the α1 helix, a network of H-bonds is present between Gln493, Gly496, Gln498, Thr500 and Asn501 from the RBD and Lys31, His34, Glu35, Tyr41, Gln42, Leu45, Lys353, and Arg357 from hACE2 (Figure 6B) [43]. Additionally, in the C-terminal portion there are H-bonds (Gln474-Gln24, Asn487-Tyr83), Van der Waals forces (Phe486- Met82) and π-stacking interactions (Tyr489-Phe28, Phe486-Tyr83), with more interactions through the middle cavity (Lys417/Tyr453 from RBD and Asp30/His34 from hACE2) (Figure 6C) [36,44].

Some structures also focus the RBD without mutations (PDB 7KMB/7A91/7A92), nevertheless they are reported in studies describing the behaviour of the entire spike protein, where the interaction region was built from non-mutated RBD focused structures (PDB 6VW1/6M0J/6LZG) [39,45].

Determining the structure of full-length human ACE2 bound to the RBD (PDB 6M17) requires the use of an amino acid transporter (B0AT1) to stabilize the protein. When bound to B0AT1, each hACE2 can bind an RBD, suggesting that native dimeric hACE2 can accommodate two S protein trimers [36] Moreover, B0AT1 is not involved in dimerization, thus hACE2 may be a homodimer even in the absence of B0AT1 [36]. Critically, the distance between the ACE2 molecules, particularly for residue D615, is only 53 Å, and thus it appears unlikely that the dimeric ACE2 can engage more than one RBD from the same spike protein without substantial conformational changes, although it could bind two RBDs from separate viral particles [46].

#### 2.1.2. Structures with Mutations

The main goal of determining the structure of a focused RBD without mutations is to understand the behaviour of the wild-type strain and to describe the main interactions of the S protein with hACE2 [43]. When introducing mutations or studying different strains, the goal may be to obtain new therapeutic agents based on either hACE2 or S protein, and to understand why new strains behave differently [23,24,41,47]. 

The four structures that focus the RBD and have mutations can be further categorized according to the aim of finding new anti-SARS-CoV-2 therapeutic agents (PDB 7DMU/7BH9), or to study mutations in new strains (PDB 7NXC/7LO4).

An hACE2 with mutations to increase the affinity to the viral protein was developed as an anti-SARS-CoV-2 therapeutic agent (PDB 7DMU), achieving more than 100-fold increase in blocking activity, when compared to the wild-type hACE2 [41]. 

The interaction pattern of engineered hACE2 with the RBD is similar to wild-type hACE2, extending a hydrophobic pocket and adding a new H-bond (K35 - Q493, from hACE2 and RBD, respectively), shortening the distance between hACE2 α1-helix to the RBD by ~1Å [41]. The use of recombinant hACE2 instead of Abs has an important advantage, given that the resulting mutants with escape mutations have decreased binding affinity to both the engineered and wild-type hACE2, leading to a decrease in virulence [41].

On the other hand, developing a competitive inhibitor from the RBD also holds potential for significant inhibition, particularly as the affinity of the inhibitor with the hACE2 is increased. The RBD inhibitor with the highest binding affinity found (PDB 7BH9) has a RSMD of 0.66 Å when superimposed with the wild type [38]. It was developed from multiple libraries of SARS-CoV-2 clones generated by random mutagenesis and selection of best expressing clones, with several cycles of mutation and testing, attempting to mimic natural evolution in vitro [38]. 

**Figure 7:**
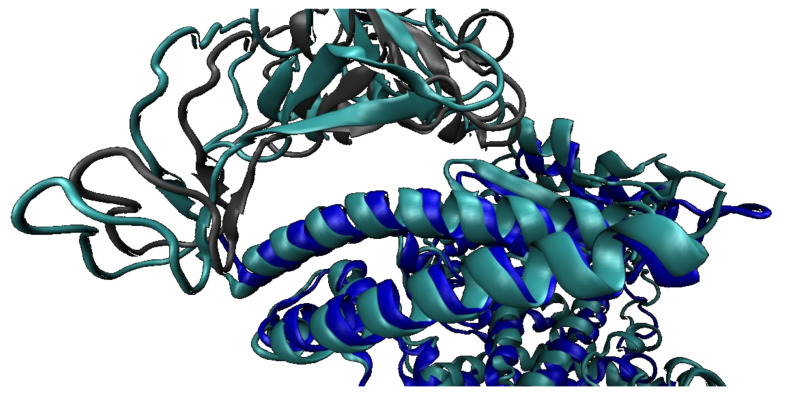
Superimposition and alignment of wild-type RBD and hACE2 (PDB 6LZG) (light blue) and mutated RBD (PDB 7BH9) (hACE2—dark blue ribbon/SARS-CoV-2 S protein—grey ribbon). The closer distance between the chains from the initial to the optimized model reflects the additional interactions established [33].

The mutated RBD (PDB 7BH9) presents, among others, eight amino acid changes actively engaged in the interaction with hACE2, namely V445K, N460K, I468T, T470M, S477N, E484K, Q498R and N501Y. These changes result in additional H-bonds, salt bridges and increased networks of interactions, both stabilizing the structure and resulting in a more positive surface, complementary to the negatively charged hACE2 interaction interface (Figure 7) [38]. The engineered protein can block viral entry and replication, due to an optimized binding, while maintaining no negative effect in the hACE2 enzymatic physiologic activity [38].

Although the optimized structure shows a possible path to design inhibitors, it is noteworthy which mutations are appearing and the sequence in which they appear. Some of the mutations present in the optimized RBD are also present in new variants as critical mutations in variants of concern, appearing often and early in the mutagenesis process, particularly mutations N501Y (variants Alpha, Beta and Gamma) and E484K (variants Beta and Gamma) [48,49,50]. This may suggest that the in vitro process is possibly mimicking natural evolution, and that artificial selection may have important predictive capabilities over new emerging mutations [41,48,49].Considering the main variants in circulation, only one mutation providing a clear survival advantage is located outside the RBD (mutation D614G, discussed in more detail ahead) [38,51]. The most common mutations appearing in the later phases, suggest that the next variants with increased infectivity might include mutation Q498R or N460K, as they both significantly increase the affinity and emerged late in the optimization process [38].

Besides predicting potential mutations, the optimized structure may also predict the effect on immune evasion. Analysis of 96 Abs revealed that 28 of them bind outside of the hACE2-binding domain, and the interaction was impaired in 9 Abs with major clashes. Additionally, mutation Q498R, besides increasing the affinity with hACE2, was frequently involved in clashes with Abs [38].

Regarding natural occurring mutations, two structures are available, a strain with mutation G485R and variant Gamma.

**Figure 8:**
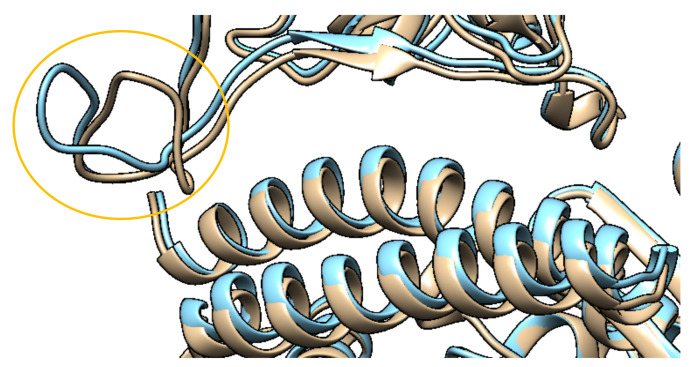
Structural superimposition between the wild-type RBD (Tan) (PDB 6M0J) and with the G485R RBD mutation (Cyan) (PDB 7LO4), with conformational change in the highlighted loop. The RMSD between structures is 0.42Å [29].

In the structure with mutation G485R (PDB 7LO4), residue 485, mutating from glycine to arginine, is not directly involved in the hACE2-SARS-CoV-2 S protein interaction, but close to Ab binding epitopes and in a loop region that makes multiple contacts with hACE2. This mutation leads to a rotation in the loop, affecting some interacting residues without significantly reducing the affinity [47]. Nevertheless, the conformational changes in the loop correlate with previous evidence that this mutation plays a role in antigenic escape from neutralising Abs, disrupting interactions involving E484 and F486 (Figure 8) [47].

**Figure 9:**
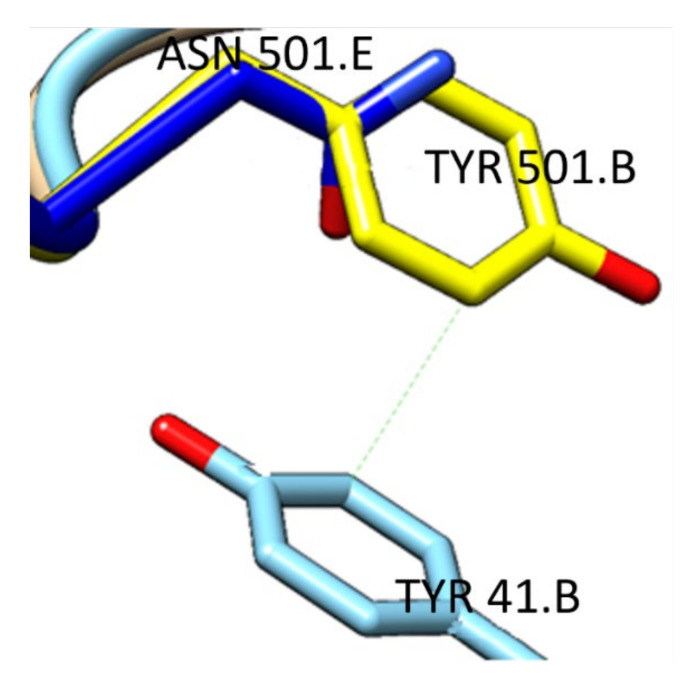
Asparagine-501 from wild-type RBD (PDB 6M0J, carbon atoms in blue) and tyrosine-501 from Gamma variant (PDB 7NXC, carbon atoms in yellow), capable of establishing an additional pi-stacking interaction with tyrosine-41 from hACE2 (bottom) [29].

Variant Gamma (PDB 7NXC), has been a cause for concern since it emerged in Brazil in the final months of 2020, showing increased transmissibility and low cross immunity [23,50]. One of the defining characteristics of this variant is the high number of mutations, particularly in the spike protein, with three mutations specific to the RBD (K417T, E484K and N501Y), that lead to a 19-fold increase in affinity compared to the Wuhan strain [50]. Mutation K417T deletes a salt bridge with D30 from hACE2, potentially leading to a decreased affinity. Critically, it hinders Ab binding, contributing to the immune escape observed with this strain [24,52]. The increased affinity is due to both mutation E484K, improving the electrostatic complementarity with the negatively charged hACE2, through the change from a carboxylic acid (E) to an amine group (K), and mutation N501Y creating an additional ring stacking interaction, with the change from the short-chained asparagine to the aromatic chained tyrosine (Figure 9) [24]. 

#### 2.1.3. RBD Only Structures—Final Remarks

The interaction interface and critical contacts between hACE2 and SARS-CoV-2 S protein are well understood. Besides having quality structures available, mutations to the RBD critical amino acids result in predictable and replicable changes in binding affinity [38,40]. When considering the differences between the wild-type RBD (PDB 6VW1/6M0J/6LZG) and the optimized RBD (PDB 7BH9), there is a path for increased binding, infectivity, and potential new variants. Some changes in the optimized RBD required two nucleotide mutations, which takes longer in natural selection [38]. Importantly, N501Y and E484K were present, emerged frequently in earlier stages and have already been found in emerging variants, suggesting some predicting potential for possible new variants [24,38]. The selection of new strains may also be related to the use of low-quality masks, as strains with a higher viral load or a tighter binding spike protein have an advantage over wild-type SARS-CoV-2 [36,38,39,44].

### 2.2. SARS-CoV-2 Full Spike Protein—hACE2 Interaction

#### 2.2.1. Mutations in the Spike Protein

In various structures of the full spike protein, some mutations appear often, namely mutation of amino acids 986 and 987 to proline and mutations in the S1/S2 cleavage site. The introduction of two consecutive proline residues, at the beginning of the central helix (residues 986 and 987), is a general strategy to retain β-coronavirus S proteins in the prototypical prefusion conformation, which facilitates the study of this conformational state [53]. The same occurs with the cleavage site (RRAR) mutation, as mutating this sequence stabilizes the prefusion structure, since cleavage activates the protein [1,28]. This cleavage site, recognized by host proteases, is fundamental for the virus, allowing the conformational changes leading to the post-fusion structure [54]. In vivo, it increases infectivity, as proteases is ubiquitously present, and importantly, this effect is present not only in SARS-CoV-2 but also in Influenza viruses and human immunodeficiency viruses [55]. Furthermore, this is a distinct characteristic of SARS-CoV-2 and absent in other β-coronaviruses, particularly SARS-CoV [55]. 

#### 2.2.2. SARS-CoV-2 Full Spike Protein—hACE2

The RBD-hACE2 focused structures highlight the main interactions leading to receptor recognition by SARS-CoV-2, and they are critical for spike protein function. Nevertheless, the spike protein has additional functions in budding and cell fusion, mainly through conformational changes caused by the interactions with hACE2 [1,28,56]. Structure determination for the full spike protein will necessarily have limitations due to its size, and usually other structures are used to build the RBD portion of the structure [39]. However, they give an important insight into the major conformational changes to the entire protein and into critical steps culminating in cell fusion and infection [39,56].

The process through which membrane fusion occurs, particularly the entry cell process, is still undefined. This seems to include the interaction between hACE2 and the spike protein, and after recognition, entry occurs via the endosomal pathway or direct cell surface interaction [45]. In both pathways, protein activation requires a protein, either cathepsin L or TMPRSS2 respectively, as full inhibition of both blocks viral entry [45]. When considering the endosomal pathway, it is important to notice the change in pH and the resulting effects in protein structure, particularly in amino acids containing multiple ionizable groups [45].

The main difference between the structures found at physiological pH (PDB 7KNE/7KNB/7KMS) and at pH 5.5(PDB7KNI/7KNH/7KNE) is the distribution of up and down RBDs. At acidic pH, no double or triple up conformations are observed, with small changes observed at pH 4.5 and pH 4.0. Starting at pH 5.5, a region in subunit S2 near the fusion peptide, rich in aspartic acid residues, undergoes a pH-dependent switch, twisting the orientation of disulphide bonds in the structure and causing RBD movement. The domain switch, caused by the pH in endosomal entry, represents an all-RBD-down locking strategy of immune evasion, that SARS-CoV-2, as other CoVs, potentially uses to avoid neutralization by RBD-up-recognizing Abs [45].

#### 2.2.3. Before and after hACE2 Binding

The S protein gradually undergoes conformational changes as hACE2 binds to the each RBD. Particularly in the first hACE2 binding event, a major conformational change is observed, with a rigid body rotation and center of mass of the S protein movement, exposing the remaining unbound RBDs, further stabilized due to the high affinity with hACE2 [45]. The steps leading from a closed and unbound S protein to a full hACE2 binding decrease the contact area between each S1 and the corresponding S2, resulting in a weaker interaction, with the conformational changes driven by hACE2 binding events [39]. These rearrangements and restructuring events in subunits S1 and S2 may have important implications in fusion activation, as the fusion peptide is preceding the S1/S2 contact area [39]. Critically, the cleavage event increases the percentage of RBDs in the up conformation from the closed trimer (PBD 7DX5) to the open timer (PBD 7DX9), increasing the ability to bind the first hACE2 [56].

Briefly, upon cleavage between S1 and S2 domains, an increase in spike trimers in open conformation is observed (unbound open trimer—PBD 7A93), followed by hACE2 binding (1 RBD bound, 2 RBD down—PBD 7A94) and a major conformational change, exposing further RBDs (1 RBD bound, 2 RBDs up - PBD 7A95/7A96) and hACE2 binding (3 RBD bound—PBD 7A97/7A98). The major conformational change and further binding weakens the S1−S2 interaction preceding the fusion peptide region, priming the S trimer for helical rearrangements in S2, required for viral−host cell membrane fusion [39]. 

The SARS-CoV-2 original viral S protein population consists mainly of RBD down structures, with S1/S2 uncleaved. The resulting tightly closed conformation of the spike protein is indicative of a conformation masking mechanism for immune evasion of SARS-CoV-2, similar to the changes found with low pH, potentially neutralizing RBD-binding Abs [39]. This feature is also described for other CoVs, such as SARS-CoV and MERS-CoV [42]. The ratio of spike proteins with up or down conformation before and after the first hACE2 binding event has a larger increase in SARS-CoV-2, indicating that the conformational masking may be more efficient [42]. 

Another important feature of CoVs, particularly regarding immune evasion, is glycosylation. The large number of N-linked glycans covering the surface of the spike protein of SARS-CoV-2, SARS-CoV and MERS-CoV could pose a challenge to antigen recognition, which thus may help the virus evade immune response [42]. However, in the residues close to the S1/S2 cleavage site, the SARS-CoV-2 trimer forms a glycan hole, a region with less glycosylation than the rest of the protein, resulting in an unhindered access by cleavage enzymes [42]. 

Taking in account all the events occurring from recognition to cell fusion, SARS-CoV-2 is overall very sensitive to the presence of hACE2, with the open state trimer existing only momentarily in the absence of hACE2 and frequently in its presence. This interaction shifts the protein population characteristics, favouring receptor binding, protein activation and the post-fusion state [42]. 

#### 2.2.4. Spike Protein Mutation D614

From all the mutations in prevalent variants by June 2021 worldwide, mutation D614G is the only one located outside the RBD, and therefore with no direct effect in affinity (PDB 7DF4), but nevertheless, causing increased infectivity and transmission [42,57]. In the closed conformation, portions near the fusion peptide, such as the subdomain 2 and heptad repeat 1, form multiple hydrogen bonds and salt bridges. From the six bonds established in this zone, four have a direct contribution from the side chain of residue D614, suggesting that this amino acid may be essential for this interaction and subsequent stabilization of the prefusion conformation [42,57]. 

In general, mutation D614G greatly disturbs the interactions between subunits S1 and S2, due to the weakened interactions established by the side chain of glycine, in comparison with aspartic acid [42,57]. As a result, the energy barrier for the conformational change is reduced and SARS-CoV-2 D614G strains have increased sensitivity to the presence of hACE2, which could be the underlying mechanism for enhanced infectivity observed in these strains [42]. On the other hand, given the increase of RBDs in the up position, these strains are more susceptible to neutralization by RBD-directed Abs [58].

#### 2.2.5. Full Spike Protein Neutralization Strategies

Soluble hACE2 has demonstrated a protective role against SARS-CoV2 infection, blocking viral entry in cell cultures and human organoids. Due to this activity, recombinant soluble hACE2 is already enrolled in several clinical trials, as a promising treatment for SARS-CoV-2 infection [59]. The use of ACE2 as a neutralizing agent can be achieved in several ways, either by increasing the affinity or engineering dimers and trimers capable of binding several RBDs. The spike protein can bind three hACE2 molecules and, as such, it is expected that a trimeric ACE2 with a linker can inhibit SARS-CoV-2. S proteins bound to one or more hACE2 have guided the design of ACE2-based viral inhibitors (PDB 7KJ2/7KJ3/7KJ4) [59]. The binding strength of constructs using a monomer, a dimer, and a trimer of hACE2, with engineered linkers, increases as expected. The trimer structure was then optimized, resulting in a trimeric ACE2 with a 200-fold increase in binding affinity, when compared with soluble wild-type hACE2, all without hindering the physiological functions of ACE2 in the angiotensin-renin system [59]. Notably, the neutralization potency of ACE2 constructs gradually increases with the size of the construct, and correlates strictly with the binding affinity, suggesting that the interaction between the RBD and hACE2 is the main component of neutralization [59]. Further improvements of these ACE2-based therapeutic candidates include modifications to enhance protein stability, to modulate peptidase activity and to increase its *in vivo* residence time in circulation [59].

An engineered ACE2-rigid-foldon, as a trimeric protein (PDB 7CT5) inhibits eight naturally occurring mutants, including D614G and seven other RBD domain mutants [46]. The inhibition of SARS-CoV-2 by the trimeric hACE2 occurs due to the simultaneous binding of all three RBD in each spike trimer, which stabilizes the viral protein in a three-RBD-up conformation and, as this was the only conformation found, it shows the striking ability of these trimers to change the conformation of the spike protein population [46]. 

#### 2.2.6. Spike Vaccine Candidate

Considering Ab potency for virus neutralizing effects, competition with hACE2 for RBD binding is a key predictor [30]. Hence, the spike protein is the prime candidate for vaccine development, in order to elicit the appearance of Abs directed at the spike protein. The S protein resulting from the coding sequence (mRNA) in the vaccine candidate BNT162b2 (PDB 7L7F), consists of a trimeric S protein capable of binding hACE2 with high affinity and structurally intact receptor binding sites. This is achieved due to a stabilization of the protein in the prefusion conformation, by substitution of residues 986 and 987 [60]. The altered spike protein induces a high functional Ab response both in mice and non-human primates (macaques), protecting them from infection with SARS-CoV-2, demonstrated by an absence of detectable RNA by RT-qPCR in the lower pulmonary tract [60].

### 2.3. Delta Variant

Spike protein mutations can be selected for either increased infectivity, generally from an increased interaction with hACE2, or decreased immune response, affecting Ab binding. As such, mutations in the spike protein are a fundamental aspect in variants of concern. Notably, one mutation is common to all prevalent strains worldwide, D614G, associated with increased infectivity, although with no direct effect to the RBD [42].

Variant Delta appeared in India in late 2020 and is becoming the dominant strain worldwide, responsible for most infections in the US, Europe, South Africa and Australia [61,62]. Importantly, this variant combines mutations affecting infectivity and potential immune escape (Table 2) [16]. Early data have shown that viral loads are 1,000 times higher with this strain, when compared with the Wuhan strain, which also contributes to increased transmission [63].

Regarding infectivity, this strain possesses mutation D614G and one mutation in the RBD, T478K. Despite no effect with direct bonds or contacts, it increases the electrostatic complementary between the proteins [65]. As such, it is hypothesised that the Delta variant emerged mainly as a result of reduced immune recognition and enhanced transmissibility nonrelated with affinity (effects from mutations D614G, P681 and D950N) [68]. Importantly, mutation N501Y is not found in the Delta variant, in contrast with all other variants of concern.Mutation N501Y results in the highest increase in hACE2 affinity conferred by a single RBD mutation [52].

Potentially fundamental for immune escape are mutations K417N, and particularly L452R, both affecting Ab binding, and occurring in the RBD [64]. L452R has also appeared in variant Epsilon identified in California, reaching a maximum of 20% of new cases in the United States, before variant Delta became prevalent [61,69]. Although no structure on the Delta variant RBD is available, a homology model of the Delta variant RBD was built, using SWISS-MODEL, and the effect of specific and new mutations can be predicted [37]. Homology modelling is a method that predicts a 3D structure based on the aminoacidic sequence, with the only requirement of an existing experimental structure with at least 30% sequence identity [70]. The genome selected to represent the Delta strain was MZ377116, retrieved from the SARS-CoV-2 Data Hub (NCBI Virus) [71].

**Figure 10:**
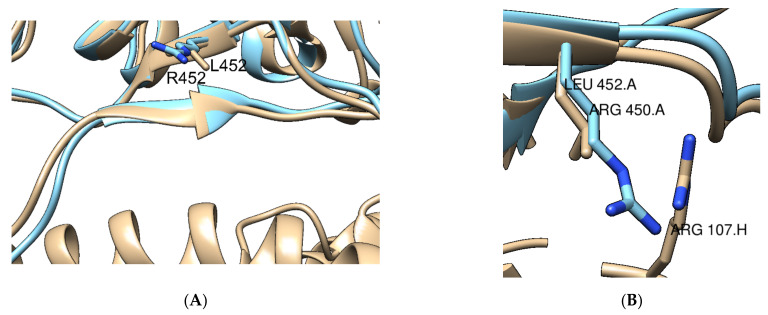
Mutation L452R, with the original variant from wild-type RBD (PDB 6M0J) (cyan) and the homology model of Delta variant (tan), obtained from genome MZ377116.1 (**A**) Superimposition of RBDs from original variant and Delta variant. Steric hindrance observed with Arg107 from Ab from structure 7CM4, due to a longer side chain in Delta variant (**B**) RBDs of original and delta variant structures with no steric hindrance regarding hACE2 [29].

Mutation L452R causes a steric hindrance to Ab binding, retrieved from structure 7CM4, without affecting binding to hACE2 (Figure 10) but causing a greater than 10-fold decrease in neutralization potency for RBD-specific Abs [61,69]. Crucially, variant Epsilon has additional mutations, S13I and W152C, resulting in severe decrease of neutralization for NTD-directed Abs, due to a new disulphide bond inducing a conformational change [64]. In the Delta variant, mutations T19R, E156-, F157- and R158G severely affect NTD directed Abs, due to the adoption of an alpha-helical conformation, instead of the original β-strand [64,68].

Variant Epsilon has been deescalated from variant of concern due the decrease in community transmission, being replaced by the Delta variant [14,16].

## 3. Conclusions

The structures available in the Protein Data Bank provide important insights into SARS-CoV-2 spike protein behaviour, clarifying the conformational changes across the protein leading to post-fusion conformation and cell infection. In general, the spike protein is highly sensitive to the presence and binding of hACE2 to the RBDs, with an allosteric effect, destabilizing the S1/S2 interaction and facilitating the final conformational change. The affinity of the complex formed in cell recognition is critical for infectivity, with consequences in the epidemiology of SARS-CoV-2. As such, the RBD is also an important target for neutralizing Abs. 

The appearance of new variants has increased SARS-CoV-2 fitness, mainly by increasing infectivity, or decreasing immune response. The Delta variant carries particular mutations associated with increased viral fitness through decreased antibody binding, increased RBD affinity and altered protein dynamics, which justifies the increased prevalence worldwide [67,72]. Despite this, some of the most common mutations increasing affinity in other variants are absent, which still show potential for evolution [52,61]. 

The ability to predict infectivity and immune escape for new variants is crucial to decrease their impact. As such, mutagenesis studies have also shown an important predicting capacity for the major mutations in current variants of concern [38]. Combining both existing mutations and mutagenesis studies, new potential SARS-CoV-2 variants, harboring advantageous S protein mutations, may be predicted. These include mutations S13I and W152C, decreasing antibody binding, N460K, increasing RDB affinity, or Q498R, positively affecting both properties 

## Figures and Tables

**Table 1:** List of variants currently or in the past considered as variants of concern or variants of interest.

WHO Label	Pango Lineage	Origin	Infectivity	Effects on Immune Response	Clinical Relevance	Current Classification (CDC/WHO/ECDC)	References
Alpha	B.1.1.7	United Kingdom Sep-2020	50% increased transmission	Minimal impact	Increased severity	VOC | VOC | DE	[14,16]
Beta	B.1.351	South Africa May-2020	50% increased transmission	Significant reduction in neutralization	Reduced susceptibility to treatment	VOC | VOC | VOC	[19,20,21,22]
Gamma	P.1	Japan/Brazil Nov-2020	Potential increased transmissibility^1^	Significant reduced neutralization	Significantly reduced susceptibility to treatment	VOC | VOC | VOC	[22,23,24,25]
Delta	B.1.617.2	India Oct-2020	Increased transmissibility	Significant reduction in neutralization	Reduced susceptibility to treatment	VOC | VOC | VOC	[14,16]
Epsilon	B.1.427/B.1.429	USA Sep-2020	Unclear	Reduced neutralization	No effect reported	NA | NA | DE	[14]
Zeta	P.2	Brazil Nov-2020	No effect reported	Potential reduced neutralization^1^	No effect reported	NA | NA | DE	[14]
Eta	B.1.525	Nigeria Dec-2020	No effect reported	Potential reduced neutralization^1^	Potential reduced susceptibility to treatment^1^	VOI | VOI | DE	[14]
Theta	P.3	Philippines Jan-2021	Potential increased transmissibility^1^	Potential reduced neutralization^1^	No effect reported	NA | NA | DE	[14]
Iota	B.1.526	USA Dec-2020	No effect reported	Reduced neutralization	Potential reduced susceptibility to treatment^1^	VOI | VOI | DE	[14,16]
Kappa	B.1.617.1	India Oct-2020	Potential increased transmissibility^1^	Potential reduced neutralization^1^	Potential reduced susceptibility to treatment^1^	VOI | VOI | DE	[14,16]
Lambda	C.37	Peru Dec-2020	Potential increased transmissibility^1^	Significantly reduction in neutralization	No effect reported	NA | VOI | VOI	[14,26]
Mu	B.1.621	Colombia Jan-2021	Potential increased transmissibility^1^	Reduced neutralization	No effect reported	NA | VOI | VOI	[14]

Abbreviations: VOC—variants of concern. VOI—variants of interest. DE—deescalated. NA- not available. Note: ^1^ Potential effect is predicted from the mutations present and their effect in previous studies. Not directly observed in the variant.

**Table 2:** List of mutations found in the spike protein of the Delta variant.

Mutation	Location	Effect on Affinity	Effect on Immune Response	Additional Information	References
T19R	S1/NTD	No effect reported	Affects NTD- directed Abs	Change in NTD antigenic site	[32,64]
E156-	S1/NTD	No effect reported	Affects NTD- directed Abs		
F157-	S1/NTD	No effect reported	Affects NTD- directed Abs		
R158G	S1/NTD	No effect reported	Affects NTD-directed Abs		
K417N	RBD	Potential decrease in affinity	Moderately decreases RBD-directed Abs	Affects only less important class of Abs	[24,52]
L452R	RBD	No effect reported	Severely affects RBD-directed Abs	None	[52,64]
T478K	RBD	Increases electrostatic complementarity	Moderately decreases RBD-directed Abs	Three additional H-bonds formed	[65,66,67]
D614G	S1-S2	No effect reported	Increases susceptibility to neutralization by RBD-directed Abs	Changes protein dynamics Increased viral loads and transmission	[52,58]
P681R	S2	No effect reported	No effect reported	Increased S1/S2 cleavage and viral fusion	[66]
D950N	S2	No effect reported	No effect reported	Change protein dynamics	[67]

Abbreviations: NTD—N-terminal domain, RBD—receptor binding domain.
